# Mechanism of action of total saponin Achyranthes in treating knee osteoarthritis explored using network pharmacology and animal experimentation

**DOI:** 10.22038/ijbms.2025.83153.17974

**Published:** 2025

**Authors:** Shiwei Zhuang, Qiang Chen, Xiao Guo, Wenhai Zhao, Ye Qiu

**Affiliations:** 1 The Third Affiliated Hospital of Changchun University of Traditional Chinese Medicine, Changchun 130117, China; 2 Institute of College of Pharmacy, Changchun University of Chinese Medicine, Changchun 130117, China; 3 Jilin Cancer Hospital, Changchun, Changchun 130012, China; 4 Affiliated Hospital of Changchun University of Chinese Medicine, Changchun 130021, China

**Keywords:** Achyranthes, Knee osteoarthritis, Anti-inflammatory, Pharmacology, Rats, Matrix metalloproteinase 9

## Abstract

**Objective(s):**

Knee osteoarthritis (KOA) is a persistent degenerative disease affecting the joints, significantly reducing the quality of life for individuals afflicted. This study explores the therapeutic effects of total saponin Achranthes (TSA) on KOA rats and its underlying mechanism.

**Materials and Methods:**

Forty-eight rats were randomly assigned to six experimental groups: a blank control group, a model group, a sham-operated group, and a TSA treatment group (low, medium, and high dose), with eight rats in each group. The rats were treated continuously for four weeks. The degree of joint swelling was quantified, and the Lequesne MG score was evaluated. Network pharmacology approaches were employed to pinpoint potential TSA targets and related pathways for managing KOA. Additionally, histopathological examinations were conducted on the knee cartilage of the rats. Serum levels of TNF-α and IL-1β were assessed through the ELISA assay.

**Results:**

The network pharmacology results indicate that TSA may effectively treat KOA through the MAPK and PI3K/Akt signaling pathways. Moreover, TSA significantly decreased the serum concentrations of pro-inflammatory cytokines such as TNF-α and IL-1β, and TSA down-regulated the P38 MAPK, PI3K/Akt, and NF-κB pathways, whereas the KOA model showed up-regulation. The treatment also significantly reduced MMP-9, MMP-13, and ADAMTS-5 protein levels.

**Conclusion:**

TSA can potentially ameliorate inflammation, safeguard knee cartilage tissue, and alleviate symptoms of KOA by inhibiting the MAPK/Akt/NF-κB signaling pathway.

## Introduction

Knee osteoarthritis (KOA) is a persistent degenerative disorder of the joints that primarily impacts older individuals and the obese population (1). The principal clinical manifestations of KOA include knee pain, swelling, impaired mobility, and end-stage deformity, which significantly impact patients’ quality of life (2). The incidence of KOA is escalating annually due to the aging global population. Global epidemiological data indicate that KOA is a significant contributor to disability (3).

Articular cartilage, a flexible connective tissue, diminishes stress and friction in synovial joints, thereby preventing joint damage (4). The compromised self-healing capacity of cartilage afflicted by illness may result in an elevated secretion of pro-inflammatory cytokines, including tumor necrosis factor-alpha (TNF-α). These cytokines and TNF-α promote the catabolic response of chondrocytes in the joints, contributing to the pathological process of KOA (5). Notably, interleukin-1β (IL-1β) is regarded as the most potent cytokine in cartilage degradation during the pathogenesis of KOA (6). It can induce articular cartilage degradation and joint inflammatory responses independently or synergistically with other cytokines. Furthermore, it enhances the synthesis of a range of inflammatory agents, including IL-6 and IL-8, stimulates the generation of reactive oxygen species (ROS), and prompts the secretion of various matrix metalloproteinases (MMPs) by chondrocytes, ultimately leading to the deterioration of the cartilage matrix and the breakdown of its structural integrity (7).MMPs comprise a group of over 25 protein hydrolases primarily secreted and acting extracellularly, regulating various biological processes by degrading protein constituents in the extracellular matrix (ECM) (8). Excessive MMP release disrupts the homeostatic equilibrium between cartilage damage and repair, leading to varying degrees of cartilage lysis and damage. Substantial evidence indicates that MMP-1, MMP-3, MMP-9, and MMP-13 play significant roles in arthritis (9). Current treatment of KOA aims to alleviate pain, ameliorate clinical symptoms, and enhance quality of life. Regrettably, long-term effective treatments are scarce for KOA. Available treatments for KOA are restricted to non-steroidal anti-inflammatory drugs (NSAIDs) and steroids (10). While NSAIDs can alleviate inflammation and pain, they are also linked with a substantial number of adverse effects, including an elevated risk of gastrointestinal, kidney, cardiovascular, or other diseases in patients (11). Hence, there is a pressing necessity to develop therapeutic drugs with fewer side effects. In recent years, clinicians and researchers have preferred traditional Chinese medicine (TCM) due to its high efficiency, low toxicity, and minimal adverse effects.TCM has demonstrated positive efficacy in treating KOA.

**Figure 1:**
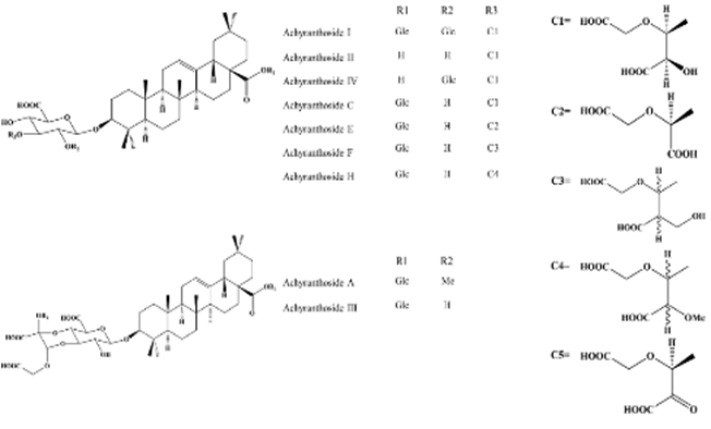
Chemical structures of nine common saponin Achyranthes

*Achyranthes refers* to the dried root of *Achyranthes bidentata* Bl. from the Amaranthaceae family. It is recognized for strengthening the liver and kidneys, enhancing muscular and skeletal strength, stimulating blood circulation, eliminating blood stasis, and promoting the downward movement of excessive inner heat. Recently, the clinical efficacy of Achyranthes has been discovered and widely applied. Such advancements have facilitated a more comprehensive comprehension of its chemical structure, pharmacological impacts, and clinical uses. Its active ingredients comprise total saponin Achyranthes (TSA),* Achyranthes bidentata *polysaccharides, and plant sterone compounds (12). The primary active ingredient of Achyranthes is TSA, characterized by oleanolic acid-type triterpenoid saponins. Figure 1 shows the structures of common Achyranthes saponins (13, 14). 

Recent pharmacological investigations have unveiled that TSA exhibits a wide range of effects, encompassing anti-inflammatory (15), anti-osteoporotic (16), anti-oxidant (17), muscle atrophy relief (18), and hepatic injury amelioration properties (19). Studies have demonstrated that TSA reduces serum IL-1β and NO levels in KOA rats. Moreover, it down-regulated the expression of MMP-3, MMP-9, and cyclooxygenase 2 (COX-2) in chondrocytes treated with IL-1β and prevented IL-1β-induced apoptosis in chondrocytes by modulating signaling pathways, including phosphorylation of p53 (15). Furthermore, Achyranthes D exhibited significant improvement in OA cartilage damage. It elevated the level of cartilage collagen II while reducing the levels of inflammatory mediators such as TNF-α, IL-1β, IL-6, and IL-18. It also inhibited the Wnt signaling pathway, leading to a reduction in chondrocyte loss and inflammation levels. This effectively alleviated KOA (20). Nevertheless, investigations into the therapeutic mechanism of TSA for KOA remain constrained.

Network pharmacology has transformed the conventional ‘single-drug, single-target’ research paradigm by adopting a holistic and systemic approach, elucidating the intricate interactions between ‘drug-gene-target’. The network topology diagram in network pharmacology enables the elucidation of the properties of traditional Chinese medicines’ actions and the mechanisms of the drug combination, thereby enhancing the scientific basis for the clinical utilization of multi-component traditional Chinese medicines. This is of tremendous importance in uncovering the intrinsic mechanism of TCM and its active ingredients and providing further research directions for traditional Chinese medicine (21). Therefore, in this investigation, we employed network pharmacology to predict the potential targets and regulate the signaling pathways of TSA for the treatment of KOA and validated it using a rat KOA model to explore the therapeutic effects of TSA on rat KOA through the p38 MAPK/Akt/NF-κB signaling pathway, to provide experimental data for the further development and utilization of TSA.

## Materials and Methods

### Plant materials and reagents

Achyranthes was obtained from Henan Province and purchased from Jiangsu Baigao Pharmaceutical Science and Technology Co. The Rat TNF-α and IL-1β Elisa kits were purchased from Proteintech(Chicago, USA). The RIPA lysis solution was obtained from Shanghai Biyuntian Biotechnology Co (Shanghai, China). Antibodies against ADAMTS5 and MMP-13 were obtained from ABclonal (Wuhan, China). Antibodies against MMP-9 were obtained from Abcam (Cambridge, UK). The remaining antibodies were obtained from Cell Signaling Technology (Boston, MA, USA). 

### Extraction of TSA

Crushed Achyranthes herbs were twice extracted with 75% ethanol (herbs to ethanol volume ratio of 1:10). The TSA was obtained by immersing the material for more than ten hours, recovering the ethanol extract, concentrating it to a specific volume at 50 °C using a vacuum rotary evaporator, and then precipitating it with 95% ethanol (to maintain the final ethanol concentration above 80%). The resulting mixture was subjected to filtration under static vacuum for ten hours at ambient temperature. Using a vacuum rotary evaporator, the filtrate was concentrated to a specific volume at 50 °C. The concentrated mixture was then dried for 24 hr in a vacuum-drying oven.

### Network pharmacology

The structural details of TSA were sourced from the PubChem database (https://pubchem.ncbi.nlm.nih.gov/). We identified potential TSA targets using the TCMSP (www.tcmsp-e.com/tcmspsearch), Stitch (http://stitch.embl.de/), and Swiss Target Prediction websites (https://pubchem.ncbi.nlm.nih.gov/). Targets pertinent to KOA were located using the Genecards database (www.genecards.org/). These targets were then standardized and validated using the Uniprot database (www.uniprot.org/) with the species set to “human.” Targets associated with TSA and KOA were visualized using Venny 2.1 (https://bioinfogp.cnb.csic.es/tools/venny/). Protein-protein interaction (PPI) networks were created by combining STRING (https://cn.string-db.org/) and Cyto-scape (version 3.9.1) software. These core targets were then analyzed using the network topology method. Furthermore, GO and KEGG pathway analyses for these core targets were performed using the Metascape database (https://Metascape.org/gp/index.html).

### Experimental animals and design

Forty-eight male SD rats, 8 weeks old and weighing between 280 and 300 g, were procured from Liaoning Changsheng Biotechnology Co (Liaoning, China). The rats were housed in an SPF-controlled environment, maintained at 24±1 °C with a humidity of 50±5%. The rats were provided unrestricted access to a standard diet and maintained under a well-ventilated light/dark cycle of 12 hr. All experimental protocols were strictly followed, following the animal care guidelines approved by the Animal Experimentation Ethics Committee of Changchun University of Traditional Chinese Medicine.

**Figure 2:**
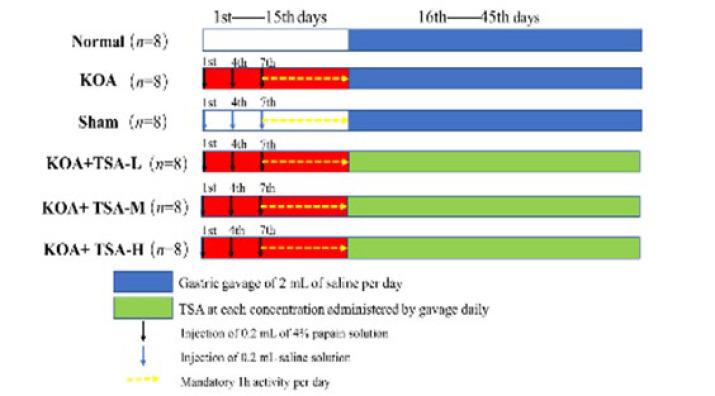
Specific steps in the in vivo experiment in rat

Following a one-week acclimation period, the 48 rats were randomly allocated into six groups: normal, model, Sham, as well as low, medium, and high dose TSA groups, with doses administered at 50, 150, and 300 mg/kg/d, each containing eight rats housed individually. The normal group received no treatment, while the other groups were modeled by intraperitoneal injection of 2% pentobarbital sodium into an anesthetized rat. With the rat anesthetized, the right hind limb was flexed at the knee joint by approximately 45°. A needle was inserted into the limb, approximately 2 mm outside and above the lower edge of the patellar ligament, with the injection direction slightly biased towards the inner aspect. The injection of 4% papain solution was then initiated; the sham group received injections of physiological saline, with a volume of 0.2 ml per injection, once every 3 days for three consecutive times (on days 1, 4, and 7) (22). From day 8 onwards, the rats were placed in an electrically operated rotating cage set to rotate at a speed of 20 rpm and engaged in activity for one hour each day. The efficacy of the modeling procedure was evaluated after eight days of activity. The presence of swelling, flexion, and extension dysfunction, as well as damage to cartilage tissues in the right knee joints of rats, were employed as indicators of successful modeling. The experimental grouping design was followed for gastric lavage treatment. Saline gavage was administered to the normal group, the KOA group, and the sham group. In the treatment group, the TSA was administered via gavage once daily for 30 days, resulting in a total of three doses. Figure 2 shows the experiment’s flow chart. After 30 days of treatment, all rats were euthanized, and serum, liver, and right knee joints were collected from each group for histopathological analysis, western blotting, and biochemical studies. 

### General behavioral observations and lequesne mg behavioral score

Throughout the experiment, we monitored general behavioral changes in each group of rats, encompassing alterations in diet, excretion, mental state, and activity. Following the final drug administration and 12 hr of fasting and water restriction, we assessed the behavioral changes of rats in each group, including responses to local pressure pain and gait. Additionally, we conducted the Lequesne MG behavioral scoring based on these observations. The scoring process was conducted by individuals who were not part of the experimental team and, thus, were not aware of the specific experimental conditions. The scores for each group were recorded. The average total score for rats in each group was calculated for comparison. The behavioral changes in the experimental rats in each group were evaluated the day following the conclusion of treatment using a modified version of the Lequesne MG Scale (23), as illustrated in Table 1.

### Histopathological examination

Liver and knee cartilage tissues were collected from the rats, fixed in 4% paraformaldehyde for 7 days, and subjected to decalcification in EDTA decalcification solution for 60 days. Subsequently, the tissues underwent dehydration through a series of graded ethanol, followed by clearing with xylene, and were then embedded in paraffin to prepare pathological sections. Following staining with hematoxylin and eosin (HE), the sections were examined under a microscope, and the pathological changes in the liver tissue and knee cartilage of rats in each group were documented.

### Saffron O-fast green staining

After complete decalcification of the tissue using an EDTA chelator, it underwent dehydration, clearing, paraffin embedding, sectioning, and deparaffinization. Subsequently, the saffron O-fast green staining method was employed to visualize the pathological alterations in the cartilage tissues under a light microscope. The resultant images were captured and analyzed.

### ELISA for serum IL-1β, TNF-α levels

Blood samples were obtained from each group on the day of surgery, and serum was separated from these samples. The concentrations of IL-1β and TNF-α in the serum were measured using ELISA assays.

### Western blot

The cartilage from the right knee joint was ground using liquid nitrogen and transferred to an EP tube. Subsequently, the tube was lysed with RIPA lysis solution containing 1% PMSF on ice for 15 min. The resulting lysate was centrifuged at 12,000 rpm to collect the total proteins mixed with the loading buffer. The protein samples underwent SDS polyacrylamide gel electrophoresis, followed by the transfer of proteins onto a PVDF membrane. The membrane was closed with 5% BSA at room temperature for 1.5 hr. The primary antibodies for MMP-9, MMP-13, ADAMTS5, p38 MAPK, p-p38 MAPK, Akt, p-Akt, NF-κBp65, p-NF-κB p65, and GAPDH, all diluted at 1:1000, were prepared in primary antibody diluent. The mixture was incubated overnight at 4 °C on a shaker. Subsequently, the membrane was incubated with a horseradish peroxidase-labeled goat anti-rabbit secondary antibody at a dilution of 1:5000 at room temperature for 1.5 hr. ECL chemiluminescent solution was then applied dropwise to the membrane, which was developed using a multifunctional gel imager. The grayscale values of the protein bands were quantified using Image J software (version 1.48u) to analyze protein expression levels.

### Statistical analysis

The statistical analysis was performed using SPSS software (version 21.0). The results are expressed as mean ± Standard Error of Mean (SEM). One-way analysis of variance (ANOVA) was used to compare differences among multiple groups. A P-value of ≤0.05 was considered statistically significant.

## Results

### Disease target collection results and intersecting targets venny plot

**Figure 3:**
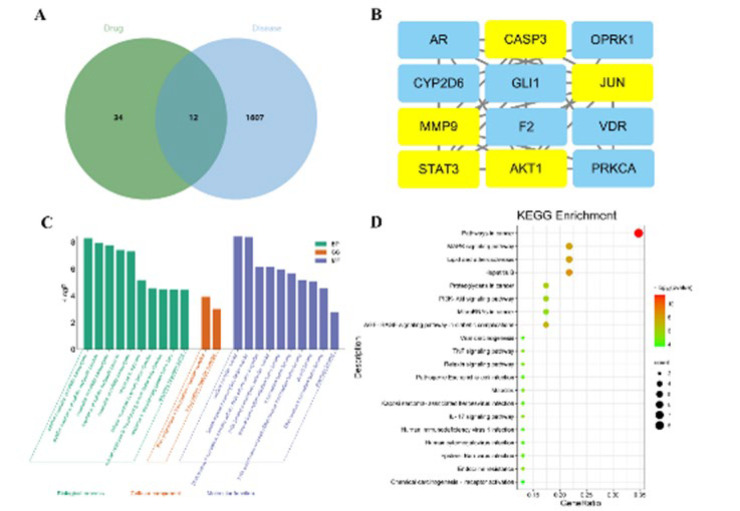
Immunosuppressive targets of TSA were analyzed using KEGG and GO analysis

The TCMSP database was employed to identify the active ingredients present in TSA. This process yielded 34 active ingredients following the elimination of duplicates. A further search of the GeneCards website for the keyword “knee osteoarthritis” yielded 1,607 disease targets. The drug and disease targets were then imported into the Venny online tool, which generated a Venny diagram illustrating the intersecting targets. This analysis yielded 12 drug-disease targets, which may represent potential targets for TSA to treat KOA (Figure 3A).

### PPI network of TSA and KOA common targets

The 12 potential targets underwent analysis in the String database to generate a target interaction network diagram. The top five targets, in descending order, are MMP9, JUN, CASP3, STAT3, and AKT1 (Figure 3B). This indicates that such targets are pivotal for TSA in treating KOA and might have a fundamental role in TSA’s approach to managing KOA.

### GO and KEGG enrichment analysis

The 149 potential action targets that were initially identified were uploaded into the String database, and a target interaction network diagram can be obtained. These genes primarily regulate miRNA transcription, response to hormones, response to growth factors, and other biological processes. They are also involved in nuclear receptor activity, ligand-activated transcription factor activity, and other molecular functions (Figure 3C). KEGG pathways were considered enriched if the *P*-value was less than 0.05. The top 20 major signaling pathways were retained, and the KEGG pathways related to KOA were mainly involved in the MAPK signaling pathway, PI3K-AKT signaling pathway, TNF signaling pathway, and other signaling pathways (Figure 3D).

### General condition and Lequesne MG behavioral scores of rats in six groups

**Figure 4:**
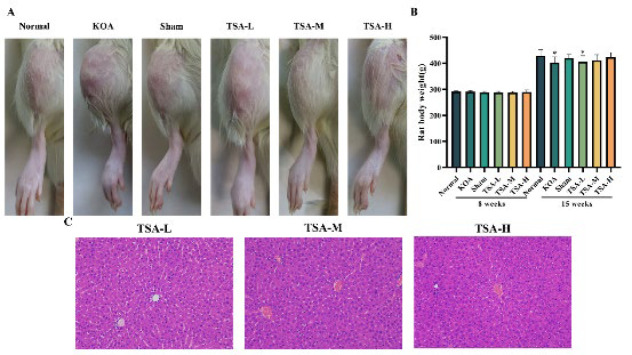
Following treatment with TSA, there was a restoration of impaired knee flexion and extension functions, a reduction in knee joint swelling, and a gradual return to normal food intake levels in rats

The rats in the normal group displayed a healthy mental state, engaged in normal activities, had smooth fur, maintained a normal diet, and displayed normal urination and defecation. Conversely, the rats in the KOA group exhibited signs of irritability and inactivity. They demonstrated limping during forced movement and dysfunction in knee flexion and extension. The rats in the Sham group exhibited similar characteristics to those in the normal group. Over time, the mental state of the TSA group gradually improved alongside the restoration of knee flexion and extension function and a reduction in bipedal limp. At the 7-week mark, rats in the model group exhibited noticeable swelling in their knee joints; however, after 30 days of TSA treatment, the swelling decreased (Figure 4A). Furthermore, their food consumption gradually returned to normal levels. The weight of the rats after 7 weeks (Figure 4B), along with the behavioral scores of the six groups, are presented in Table 2.

The results of HE staining revealed a clear and intact structure of the liver tissue in the TSA group rats at the experiment’s conclusion. The nuclei of the cells exhibited uniform and distinct staining, devoid of edema or inflammatory cell infiltration. These findings suggest that oral TSA administration does not induce liver damage or hepatotoxicity (Figure 4C).

### Effect of TSA on serum inflammatory factor levels in rats with KOA

**Figure 5:**
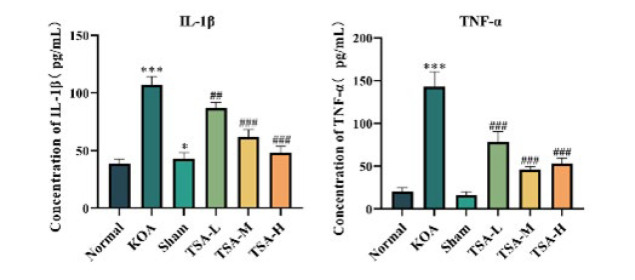
Effect of TSA on serum IL-1β and TNF-α levels in rats

In the KOA group, serum IL-1β and TNF-α levels were significantly elevated compared to the normal group (*P*<0.001). While serum IL-1β levels were significantly elevated in the Sham group, TNF-α levels did not exhibit a significant alteration (*P*>0.05). The serum levels of IL-1β and TNF-α were significantly reduced in the rats of the TSA-L, TSA-M, and TSA-H groups compared to the KOA group (*P*<0.01; *P*<0.001), as depicted in Figure 5.

### Effects of TSA on ADAMTS-5, MMP-9 and MMP-13 proteins in a rat KOA model

**Figure 6:**
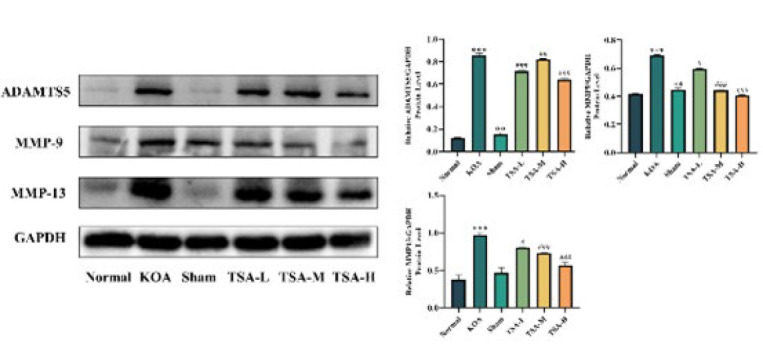
Effect of TSA on ADAMTS5, MMP-9, and MMP-13 protein expression levels in rat knee cartilage

Figure 6 illustrates that the protein **expression levels of MMP-9,** MMP-13, and ADAMTS-5 were significantly elevated in the KOA group compared to the normal group (*P*<0.001). The protein expression level of MMP-13 remained unchanged in the Sham group (*P*>0.05); however, following treatment with each dose of TSA, the protein expression levels of **MMP-9**, MMP-13, and ADAMTS-5 significantly decreased compared to the KOA group (*P*<0.05, *P*<0.01, and *P*<0.001). 

### TSA treatment alleviates the joint pathological changes caused by KOA

**Figure 7:**
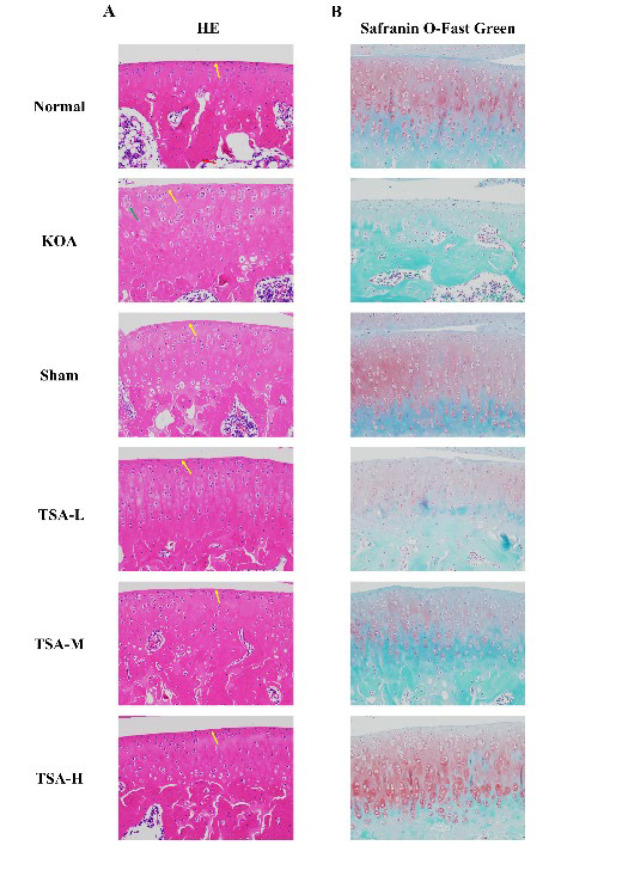
TSA ameliorates KOA development in rat KOA model *in vivo*

To assess TSA’s protective effect on KOA progression *in vivo*, we induced KOA in SD rats by intra-articular injection of papain. HE staining results revealed that knee cartilage tissues in both the normal and sham groups exhibited a normal overall structure. The cartilage surface was smooth, and the cellular demarcation of cartilage at various levels was clear. No obvious detachment of articular chondrocytes or fibrosis of articular cartilage was observed. In the KOA group, the articular cartilage, indicated by yellow arrows, exhibited abundant trabeculae without apparent fractures. The trabeculae, highlighted by red arrows, showed no signs of inflammatory cell infiltration. Compared to the normal group, the KOA group exhibited mild abnormalities in the general structure of cartilage tissue. The cartilage surface in the KOA group appeared rough, with observed fibrosis on the surface layer (highlighted by yellow arrows in the figure). Furthermore, chondrocyte proliferation (indicated by green arrows in the figure) and tissue infiltration by inflammatory cells were evident. The cartilage tissue displayed a normal overall structure with a smooth surface. It was observed that there was a clear demarcation of cellular structures at various levels of cartilage, with no evidence of inflammatory cell infiltration in the tissue (Figure 7A).

The saffron O-fast green staining results demonstrated that the cartilage surface of both the normal and sham groups exhibited a smooth and intact appearance with no evident erosion defects. The chondrocytes exhibited normal morphology and distribution, characterized by uniform matrix staining and a complete, continuous, and clear tide line. Within the model group, larger areas of cartilage erosion defects were observed, accompanied by a serious local rupture and detachment phenomenon. Furthermore, there was a significant reduction in cartilage matrix staining. With increasing administered dose, cartilage surface erosion damage decreased gradually in the TSA-L, TSA-M, and TSA-H groups, while cartilage matrix staining significantly increased. The TSA-H group demonstrated significant improvement, characterized by neatly arranged chondrocytes, clear boundaries between cartilage and subchondral bone, uniform staining, and evident tide lines (Figure 7B).

### Effect of TSA on protein expression of p-p38MAPK, p-Akt, and p-NF-κB p65 in rat knee cartilage

**Figure 8:**
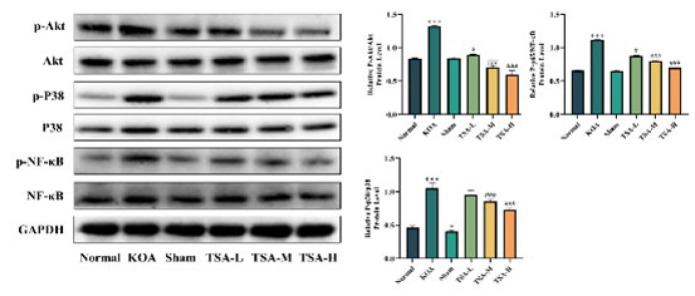
Effect of TSA on NF-κB p65, p38 MAPK, and Akt phosphorylation

The progression of KOA is associated with the production of pro-inflammatory cytokines. Overexpression of these cytokines is typically triggered by the p38 MAPK/NF-κB and PI3K/Akt/NF-κB signaling pathways. To assess the activation of the p38 MAPK, Akt, and NF-κB pathways in KOA rats, western blotting analysis was conducted, as depicted in Figure 8, the grayscale ratios of p-p38/p38, NF-κB/p-NF-κB, and p-Akt/Akt in the KOA group were significantly higher compared to the normal group (*P*<0.001), indicating phosphorylation of p38, NF-κB, and Akt. Greyscale values of p-p38/p38, NF-κB/p-NF-κB, and p-Akt/Akt were significantly lower in all TSA groups compared to the KOA group (*P*<0.05 and *P*<0.001). These results indicate that TSA could alleviate the inflammatory response by inhibiting the phosphorylation of p38, NF-κB, and Akt, thereby suppressing the p38 MAPK/Akt/NF-κB signaling pathway.

## Discussion

KOA is a degenerative condition characterized by knee pain, swelling, and functional disability, significantly impacting patients’ quality of life. The precise etiology of KOA remains elusive, although it is commonly associated with several factors, including genetics, inflammation, obesity, sports injuries, and gender (24). The diagnosis of KOA in its early stages can be achieved by identifying clinical symptoms and utilizing magnetic resonance imaging (MRI)(25). Currently, treatment options for KOA include surgical and pharmacological interventions, which aim to alleviate clinical symptoms and delay disease progression. However, it should be noted that these treatments do not offer a complete cure. Moreover, numerous drugs are associated with adverse effects (24). Consequently, identifying an effective treatment modality for KOA that minimizes adverse effects holds significant clinical importance.

KOA primarily affects individuals in the middle to older age groups. According to traditional Chinese medicine, the aging process is linked to a deficiency of qi and blood in the liver and kidneys, which may lead to the infiltration of harmful factors. A prolonged illness frequently reduces the circulation of qi and blood, which can lead to pain. Achyranthes, an herbal remedy employed in traditional Chinese medicine, nourishes the liver and kidneys, strengthens tendons and bones, improves blood circulation, and alleviates pain (26). Its primary active component, TSA, is anticipated to exhibit preventive and therapeutic effects on KOA. This research aimed to assess the efficacy of TSA for treating KOA and to elucidate its underlying mechanism. Nevertheless, the precise pharmacological mechanism of TSA in managing KOA with Achyranthes is still not well-defined. To address this gap, we utilized a network pharmacology strategy to analyze the interactions among active ingredients, their targets, and associated signaling pathways. Subsequently, an experimental approach was employed to investigate the possible mechanisms through which TSA operates. The findings indicated that AKT1 and MMP9 were the primary targets for treating KOA with TSA. The treatment of KOA with TSA primarily involved the MAPK, PI3K-Akt, and TNF signaling pathways.

Papain, an enzyme that breaks down proteins, is used to degrade proteoglycans in the cartilage to induce KOA in rats. This degradation process leads to the liberation of chondroitin sulfate and the generation of pro-inflammatory factors, which reduces the protective function of chondrocytes. By injecting papain directly into the knee joint cavity, KOA-like symptoms similar to those observed in humans can be induced in rats (27). Therefore, this study effectively created a rat model of KOA by injecting a solution containing 4% papain into the right knee joint cavity, thereby altering the joint cavity’s microenvironment and simulating daily activities. This method offers the advantages of a short modeling time and ease of operation. The results showed that rats in the KOA group exhibited symptoms such as reduced activity, limping, impaired knee flexion and extension, and weight loss. The group treated with three doses of TSA exhibited improvement in the preceding conditions.

In the early stages of KOA, the cartilage undergoes focal softening and loss of elasticity. Subsequently, it progresses to roughness, erosion, ulceration, and even extensive detachment and disappearance of the cartilage (2). The study examined the degeneration and destruction of rat knee joint cartilage following papain induction through visual observation, HE staining, and saffron O-fast green staining. The KOA group exhibited the most severe cartilage damage. Increasing the dose of the TSA drug significantly improved the surface damage, color, and morphology of knee joint cartilage in rats. Furthermore, the drug effectively alleviated surface folds, breakage, tidal line defects, staining loss, and uneven staining of the upper and lower layers of cartilage. Histopathological examination of cartilage in KOA rats also revealed improvement.

Individuals with KOA frequently exhibit impaired inflammatory responses. Research has demonstrated the critical roles of TNF-α and IL-1β in pain during the early stages of KOA, correlating with pain (28). IL-1β plays a pivotal role in the development of osteoarthritis, capable of triggering inflammatory reactions alone or in conjunction with other mediators, affecting the joint’s articular cartilage and other structural components. In the development of KOA, there is a marked increase in the infiltration of inflammatory cells into the synovial tissues and enhanced disruption of cartilage due to elevated IL-1β levels (29). The pro-inflammatory effects of TNF-α and IL-1β have been established. Throughout OA progression, these cytokines attach to specific receptors, stimulating the release of more pro-inflammatory cytokines like IL-6 and IL-8, mediated through NF-KB and MAPK pathways. The article reports up-regulation of ADAMTS-5, NO, and PGE2 expression levels alongside increased MMP-1, MMP-3, and MMP-13 content in chondrocytes and synovial fibroblasts. This leads to an enhanced inflammatory response, degradation of the extracellular matrix, inhibition of extracellular matrix synthesis, and ultimately, degeneration, destruction, and degradation of articular cartilage (30). Additionally, the examination of TNF-α and IL-1β concentrations in rat serum revealed no significant changes between the Sham and normal groups. This suggests that the injection method did not affect the organism. Conversely, serum levels of TNF-α and IL-1β significantly decreased following TSA treatment compared to the KOA group. This suggests that TSA can inhibit the manifestation of inflammatory factors, thereby suppressing joint inflammation and controlling the degeneration of articular cartilage in KOA. 

The research further delves into TSA’s mechanism through a network pharmacology analysis. The protein-protein interaction (PPI) network analysis results indicate that the key target genes are MMP9, JUN, CASP3, STAT3, and AKT1. In addition, MMP-9, a critical enzyme linked with Sdc4 in OA conditions, potentially reduces chondrocyte sensitivity to IL-1β signaling. MMP-9 is responsible for the degradation of most components within the cartilage matrix, including collagen and proteoglycans. This process accelerates the catabolism of the cartilage matrix, which in turn leads to the gradual apoptosis and necrosis of chondrocytes. This results in the exacerbation of the pathological process of KOA, as well as accelerated inflammation (31). The KEGG enrichment pathway analysis results indicated that the TSA treatment of KOA is primarily associated with the MAPK signaling pathway, PI3K-AKT signaling pathway, TNF signaling pathway, and IL-17 signaling pathway. The pathways described above are intimately linked to the pathogenesis of KOA.

The MMP family includes collagenases 3, 9, and 13, the most active enzymes. They play a pivotal role in the degradation of the ECM. The expression of MMP-13 is strongly correlated with the severity of the lesion, making it a valuable indicator of disease progression. MMP-13 is a highly active protease abundantly expressed in articular cartilage and chondrocytes. It specifically degrades type II collagen and proteoglycans(32).ADAMTS-5, another proteoglycanase, cleaves proteoglycans and also contributes to cartilage damage in osteoarthritis (33). The study found significantly higher MMP-9, ADAMTS5, and MMP-13 expression levels in the cartilage of rats with KOA. The results indicated that TSA effectively suppressed the abnormally elevated expression of MMP-9, ADAMTS5, and MMP-13 in the cartilage of rats with KOA, improved chondrocyte apoptosis and extracellular matrix degradation, and protected the articular cartilage. The protective effect was even more pronounced at higher doses.

P38 is a crucial member of the MAPK family that regulates the inflammatory response. Upon activation, p38 MAPK translocates to the cell nucleus. It acts as a downstream signaling molecule, enhancing IκBα phosphorylation and degradation, thus activating the NF-κB pathway (34). This activation is crucial in regulating KOA through NF-κB signaling, primarily through the p-NF-κB form, contributing to the release of cytokines such as IL-1β, IL-6, and TNF-α (35). Inhibiting the activation of the NF-κB signaling pathway can mitigate the release of these inflammatory factors, slowing down the process of cartilage degeneration and KOA. Meanwhile, NF-κB phosphorylation could impact MMP9 activation(36), corresponding to targets predicted by network pharmacology. The experimental findings demonstrate that TSA inhibits the p38 MAPK and NF-κB pathways, as evidenced by the protein expression levels of p-p38 MAPK/p38 MAPK and p-P65 NF-κB/P65 NF-κB. This suggests that TSA may exert a therapeutic effect on KOA rats by diminishing the production of inflammatory cytokines by inhibiting the p38 MAPK/NF-κB pathway. 

Research has demonstrated a close association between KOA and the PI3K/Akt pathway (37, 38). Activating this pathway can increase MMP synthesis through various downstream targets, with NF-κB as a key regulator. Stimulation of cytokine receptors triggers the membrane protein PI3K to induce AKT phosphorylation, subsequently activating NF-κB (39). Therefore, inhibiting the PI3K/Akt pathway can attenuate cartilage degradation and inflammatory responses in KOA rats. This study found that TSA effectively inhibited Akt phosphorylation and NF-κB activation, thereby reducing cartilage damage.

In summary, the network pharmacological approach to TSA for treating KOA is characterized by multi-target and multi-pathway. This effect may be exerted by reducing the expression of TNF-α, IL-1β, MMP-9, MMP-13, and ADAMTS-5, as well as inhibiting the p38 MAPK/Akt/NF-κB signaling pathway in the knee cartilage tissues. Therefore, TSA holds promise as a therapeutic agent for managing diseases related to KOA. However, due to the complex pathogenesis of KOA, further investigation is required to determine if other pathways are involved in regulating cartilage damage by TSA.

The normal group was not subject to any intervention, whereas the remaining five groups were modelled by the injection of a 4% papain solution into the knee joint cavity, followed by forced activity. After 30 days of treatment, all rats were euthanized, and serum, liver, and right knee joints were collected from each group for histopathological analysis, western blotting, and biochemical studies.

**Table 1:** Improved version of the knee osteoarthritis severity index

Scoring items/points	0 point	1 point	2 point	3 point
Local pressure on the affected knee to observe the degree of pain stimulus response	No abnormal pain response	Contraction of the affected limb occurs	Contraction of the affected limb with mild systemic reaction	Violent contractions of the affected limb with generalized trembling and struggling
Repellent observation gait	No limp in the affected limb, strong stirring of the ground	Mild limp, strong stirrups	Participation of the affected limb in walking with marked lameness	Affected limb does not participate in walking and cannot stir the ground
Joint range of motion	>90°	45°–90°	15°–45°	<15°
Swelling of the joints	No swelling	Mild swelling	Obvious swelling	----

(A) Venn diagram of active ingredients and disease targets. (B) PPI network of common targets of TSA and KOA. (C) GO functional enrichment analysis. (D) Analysis of KEGG pathway enrichment. TSA: Total saponin Achyranthes; KEGG: Kyoto encyclopedia of genes and genomes; GO: Gene ontology; PPI: Protein-protein interaction networks; KOA: Knee osteoarthritis

Notably, oral administration of TSA did not result in liver injury or hepatotoxicity. (A) Appearance of the knee joint was observed in six groups of rats. (B) Body weight of rats in each group was recorded at 8 and 15 weeks (n = 8). Data are the means ± SEM. Compared with the Normal group, *P<0.05. (C) Effect of TSA on the pathological morphology of rat liver (HE staining, 200×). TSA: Total saponin Achyranthes

**Table 2:** The following table presents the results of the behavioural scores of the rats in each group. These scores are based on the following observations: localised pressure on the affected knee to observe the level of pain stimulus response; repulsion to observe gait; range of joint movement; and joint swelling. It is evident that an elevated score is indicative of a more severe injury

Groups	n	Behavioral scores
Normal	7	0.00±0.00
KOA	7	6.43±1.13^***^
Sham	7	0.57±0.53
TSA-L	7	2.29±1.25^###^
TSA-M	7	2.14±1.35^###^
TSA-H	7	2.00±0.58^###^

Data are the means ± SEM. Compared with the Normal group, ***P<0.001. Compared with the KOA group, ###*P*<0.001.

The data are the means ± SEM. Compared with the Normal group, *P<0.05,***P<0.001; compared with the KOA group,##*P*<0.01,###*P*<0.001. TSA: Total saponin Achyranthes; KOA: Knee osteoarthritis

The data are the means ± SEM. Compared with the Normal group, **P<0.01 and ***P<0.001; compared with the KOA group, #*P*<0.05,##*P*<0.01, and ###*P*<0.001. TSA: Total saponin Achyranthes; KOA: Knee osteoarthritis

(A) H&E staining of knee cartilage of rats in different experimental groups after 4 weeks of treatment (magnification, 200×). (B) Saffron O-fast green staining of knee cartilage in rats from different experimental groups after 4 weeks of treatment (original magnification, 200×). TSA: Total saponin Achyranthes; KOA: Knee osteoarthritis

The data are presented as mean ± SEM. Compared with the Normal group,*P<0.05, ***P<0.001; and in comparison with the KOA group, #*P*<0.05, ###*P*<0.001.

TSA: Total saponin Achyranthes; KOA: Knee osteoarthritis

## Conclusion

TSA ameliorates knee cartilage damage in rats with KOA induced by papain. This improvement could be attributed to the decreased expression of TNF-α, IL-1β, MMP-9, MMP-13, and ADAMTS-5, as well as the inhibition of the p38 MAPK/Akt/NF-κB signaling pathway in knee cartilage tissues. These findings imply that TSA presents potential as a preventative and therapeutic intervention for KOA. Nevertheless, additional, comprehensive research and validation of the specific links in the pathways involved are still necessary. This investigation lays an experimental foundation for the clinical management of KOA with TSA and a theoretical structure for the advancement and application of TSA.

## Data Availability

Data will be made available upon request.

## Declaration

The authors applied deepl in their writing to improve the readability of the paper.
